# Connexel visualization: a software implementation of glyphs and edge-bundling for dense connectivity data using brainGL

**DOI:** 10.3389/fnins.2014.00015

**Published:** 2014-03-04

**Authors:** Joachim Böttger, Ralph Schurade, Estrid Jakobsen, Alexander Schaefer, Daniel S. Margulies

**Affiliations:** ^1^Max Planck Research Group for Neuroanatomy & Connectivity, Max Planck Institute for Human Cognitive and Brain SciencesLeipzig, Germany; ^2^MEG and Cortical Networks Unit, Max Planck Institute for Human Cognitive and Brain SciencesLeipzig, Germany; ^3^Department of Neurophysics, Max Planck Institute for Human Cognitive and Brain SciencesLeipzig, Germany; ^4^Department of Neurology, Max Planck Institute for Human Cognitive and Brain SciencesLeipzig, Germany

**Keywords:** functional connectivity, connectome, visualization software, neuroanatomy, magnetic resonance imaging

## Abstract

The visualization of brain connectivity becomes progressively more challenging as analytic and computational advances begin to facilitate *connexel-wise* analyses, which include all connections between pairs of voxels. Drawing full connectivity graphs can result in depictions that, rather than illustrating connectivity patterns in more detail, obfuscate patterns owing to the data density. In an effort to expand the possibilities for visualization, we describe two approaches for presenting connexels: *edge-bundling*, which clarifies structure by grouping geometrically similar connections; and, *connectivity glyphs*, which depict a condensed connectivity map at each point on the cortical surface. These approaches can be applied in the native brain space, facilitating interpretation of the relation of connexels to brain anatomy. The tools have been implemented as part of *brainGL*, an extensive open-source software for the interactive exploration of structural and functional brain data.

## 1. Introduction

The term *connexel* was first introduced to describe the basic unit in brain connectomics—the relationship between two three-dimensional (3D) positions (Worsley et al., 1998). As pixels are points in a 2D image, and voxels are points in 3D space, the connection between two voxels can be described as a single point in a 6D space. Connexels are modality-independent, as they can describe the relationship between pairs of voxels as assessed using any data type. However, they are particularly well suited for “pathless” methodologies that solely describe the weight of a connection between two points. This includes functional connectivity using functional magnetic resonance imaging (fMRI) data (Biswal et al., 1995) and magnetoencephalography (MEG) (Brookes et al., 2011) measurements, similarity matrices of probabilistic tractography (Johansen-Berg et al., 2004), and cortical thickness-based covariance (Lerch et al., 2006; Alexander-Bloch et al., 2013; Bernhardt et al., 2013), where the connexel captures all known information. The resulting data are still highly complex, since connectivity can be calculated between every pair of gray matter locations in the brain. This complexity makes their visualization and exploration challenging.

While interactive software for the visualization of structural connectivity is well developed, the visualization of pathless connectivity remains largely bound to technical standards developed within the task-based fMRI literature (Margulies et al., 2013), drastically reducing the complexity of the data during the visualization process. For example, *seed-based* approaches (Biswal et al., 1995) show the connectivity from a single region-of-interest. Similarly, *principle and independent component analysis* (PCA/ICA)-based methods (Beckmann et al., 2005; Damoiseaux et al., 2006; De Luca et al., 2006) reduce the data to a set of large-scale networks, which are usually displayed in separate images.

While graph theory approaches have been integrated into brain imaging methods (Bullmore and Sporns, 2009; Sporns, 2013) with a continuously developing toolbox of analytic techniques (Fornito et al., 2013), the current methods for visualization of connectivity fail to adequately represent the high dimensionality and resolution of human brain data (Margulies et al., 2013). Attempts have been made to visualize functional connectivity data by presenting a global view of brain connectivity (McGonigle et al., 2011; Irimia et al., 2012; van Dixhoorn et al., 2012; Zuo et al., 2012). However, in these cases the brain is mapped to an abstract layout that changes spatial relations and separates network structure from the underlying anatomy. Although tools have emerged to address the specific challenges of embedding network graphs in the cerebral topography (e.g., *BrainNet Viewer*, Xia et al., 2013), such approaches require the data to be reduced to a proportionally small set of regions-of-interest, which are then depicted using “ball-and-stick” methods (e.g., Worsley et al., 2005; Xia et al., 2013). These methods do not offer the possibility of depicting connexels in the brain's native 3D space at full resolution, since simply presenting all connexels as straight line segments renders the image cluttered and unreadable.

Interactive software alleviates the drawbacks of static visualizations by offering real-time display while manipulating a seed region (Cox, 1996; van Dixhoorn et al., 2010; Böttger et al., 2011; Eklund et al., 2011; Saad and Reynolds, 2012). The visualization tool *BrainCove* (van Dixhoorn et al., 2012) uses multiple synchronized views with different levels of abstraction and selection techniques. However, such software still requires the user to iteratively focus on limited aspects of the data at any given moment, much like exploring a dark room with a searchlight.

The currently available software for the visualization of connexel data provides a multitude of sophisticated tools for the visualization of connectivity. We refer to (Margulies et al., 2013) for a review of visualization techniques for connectivity, and to (Xia et al., 2013) for a compilation of software for graph-based connectivity visualization. In Table 1, we have compiled an overview of software tools relevant for the visualization of connexels. None of these tools offer a way to visualize the data at full resolution in the native anatomical space without assumptions that reduce the data, either in resolution, or because it is only possible to show connectivity from one point or region at a time. Rather than reduce the connectivity information to accommodate limitations in the space of visualization, we propose two methods specific to this technical challenge that emphasize features of the connexel-space in relation to cortical anatomy.

**Table 1:** **Overview of software for connexel visualization**.

**Software**	**Website**	**Description**	**Advantages**	**Shortcomings**
Fubraconnex	code.google.com/p/fubraconnex	C++-based functional connectivity viewer	Multiple abstract and anatomical layouts	Restricted to relatively low resolution
Connectome viewer	cmtk.org/viewer	Python-based connectivity visualization	Ball-and-stick graph visualization of connectivity	Restricted to relatively low resolution
Connectome workbench	www.humanconnectome.org/connectome	Connectivity visualization based on Caret	Fully interactive anatomical surface visualization with full resolution	Only one seed point at a time
Brainnet viewer	www.nitrc.org/projects/bnv	Matlab toolbox for brain network visualization	Ball-and-stick graph visualization of connectivity	Restricted to relatively low resolution
Visualconnectome	code.google.com/p/visualconnectome	Matlab toolbox for brain network analysis and visualization	Ball-and-stick graph visualization of connectivity	Restricted to relatively low resolution
MNET	neuroimage.yonsei.ac.kr/mnet	Matlab toolbox for brain network analysis and visualization	Ball-and-stick graph visualization of connectivity, hierarchical edge bundling, abstract and anatomical views	Edge-bundling restricted to abstract circular layout, relatively low resolution
Braincove	bitbucket.org/avandixhoorn/braincove/src	C++-based voxel-wise functional connectivity visualization	Volume rendered functional connectivity networks in full resolution	Only one seed point at a time
SUMA	afni.nimh.nih.gov/afni/suma	C++-based surface connectivity visualization	Fully interactive anatomical surface visualization with full resolution	Only one seed point at a time

The table includes existing software for the display of pathless connectivity, and their major advantages and shortcomings with respect to the aims of the current article.

### 1.1. Edge-bundling

A technique first applied to abstract hierarchical data such as call graphs for software systems (Holten, 2006) has recently shown promise for clarifying bundles of connexels by grouping geometrically similar edges (Bottger et al., 2013). Placing connections at the focus of the image produces an overview of network structure in the anatomical space, but also reduces the ability to assess its relation to cortical anatomy.

### 1.2. Connectivity glyphs

In order to clarify the anatomical position of the connections' termination points, we present for the first time the *connectivity glyph*, a small iconic display of multivariate connexel information at each location in the rendering.

We describe our integration of edge-bundling and connectivity glyphs in the open-source software *brainGL*^1^. Initially designed for the interactive exploration of structural connectivity, the software provides a framework for the manipulation and rapid interactive display of complex brain data using graphics hardware shaders. For the purposes of illustrating the visualization and exploratory applications of edge-bundling and connectivity glyphs, we present examples using functional connectivity data.

## 2. Methods

### 2.1. Programming environment

brainGL is implemented in C++, and uses the portable Qt framework^2^, which provides graphical user interface (GUI) elements, data structures, and OpenGL^3^ for hardware-accelerated rendering. Other external dependencies include GLEW^4^, Visualization Toolkit (VTK)^5^ and boost^6^. The software requires advanced features from OpenGL (version >3.3) for the efficient display of complex geometry using graphics hardware, and runs on Linux, Windows and MacOS platforms.

### 2.2. Installation and use

The installation and use of the software is described on the documentation page at: code.google.com/p/braingl/wiki/Main. We provide a precompiled binary for Windows. For Linux-based environments, the source code has to be compiled as described at: code.google.com/p/braingl/wiki/Installation. The compilation from source code requires several external libraries to be installed: boost, Qt4, VTK and GLEW. The libraries can be installed from the terminal using a package manager such as apt-get or the software center GUI in ubuntu, by searching for the following libraries: libboost, libglew, libqt4, libvtk5. The libraries and the development files (packages ending with -dev) are necessary. A further prerequisite is the installation of a cmake-based build system with a C++ compiler.

We also provide tutorials for the interactive use of the software for the viewing of connectivity glyphs (code.google.com/p/braingl/wiki/GlyphTutorial), and bundling of connections (code.google.com/p/braingl/wiki/ConnectionBundling).

### 2.3. Data formats

Displaying connexel data in brainGL requires (1) an *anatomical coordinate space* on which to present (2) *connectivity information*. Connectivity information can be created from volume time-series or group morphological data, or loaded from binary files that represent connectivity matrices. Graph representations in 3D space can either be derived from the connectivity data, or loaded using binary (.fib) or ascii (.cxls) files.

#### 2.3.1. Anatomical coordinate space

A crucial feature of brainGL is the visualization of high resolution connectivity data in relation to the underlying anatomy. The coordinate system used in the software is established by the first input volume, which is loaded from a NIFTI-format file^7^. The header information from loaded volumes is used to enable the display of volumes with differing voxel sizes. Note that the volumes have to be aligned, since any rotational component of the transformations in their headers is ignored. In addition, cortical surface meshes, as created by software packages such as FreeSurfer^8^, can be loaded as FreeSurfer ASCII-files (.asc) or VTK-files (.vtk).

#### 2.3.2. Surface connectivity matrices

The display of surface connectivity glyphs in brainGL requires a surface representation (with potentially multiple spatial representations, for example pial, inflated or spherical representation), and a full connectivity matrix between its nodes. It is also possible to display connectivity glyphs between arbitrary spatial positions, for example on slices through a volume, by loading a list of connexels as described in section 2.3.4.

The required data can be obtained from a four-dimensional NIFTI-file that contains a set of volumes, either over time or across individuals. Correlation between the series of values in the fourth dimension of a pair of voxels can then be used to establish connectivity measures. Such measures can be based on the correlation of time-series from fMRI measurements, or group-level covariance of anatomical morphometry data. After loading the 4D-volume and a registered surface mesh, a connectivity matrix can be created using brainGL by sampling the volume data to the surface nodes.

Alternatively, it is possible to directly load such data using what we call a *glyphset* file structure, which consists of three parts:

- A set of surfaces in FreeSurfer ASCII format described in a text file which we call a *set file*
- A binary file with the full square symmetric connectivity matrix, containing the connectivity values between all pairs of nodes on the FreeSurfer surfaces
- A file in ASCII format containing the filename of the set file and the connectivity matrix—this text file also specifies the lowest connectivity value included in the glyph visualizations (see section 2.3.3), and has the file ending *.glyphset*.

#### 2.3.3. Thresholding

While we do not reduce the resolution of the connectivity data for display, we enable thresholding for two reasons: (1) the total number of connexels in a full connectivity matrix can be too large for display on current graphics hardware for high data resolutions and (2) thresholding can also limit the influence of less significant connections on the resulting visualizations. Random field theory (Worsley et al., 1998), network-based statistic (NBS) (Zalesky et al., 2010) and spatial pairwise clustering (SPC) (Zalesky et al., 2012b) have been proposed to find a statistical threshold with controlled error rates. Several accepted methods are widely used for voxel-wise analyses (Nichols and Hayasaka, 2003). However, the calculation of an optimal threshold with the right mixture of specificity and sensitivity for connexels is an open research question.

For these reasons, we have adopted the following strategy in order to include as much information as is computationally feasible. Starting with the full weighted connectivity matrix as an input, only a low *minimum threshold* at load-time is applied to make fluent interaction with the glyph visualizations feasible. Using subsequent interactive thresholding during visualization, it is then possible to set minimum connectivity values and a minimum distance between connexel endpoints. While this does not resolve the problems of statistical inference, it is beneficial for the exploration of datasets without additional assumptions.

The edge-bundling algorithm is more computationally restricted owing to: (1) the use of binary graphs in the current implementation, and (2) the complexity of the method, which makes it applicable only for a smaller number of connexels than those rendered as connectivity glyphs. While an adaption to weighted graphs can potentially enable bundling for full connectivity graphs in the future, our current implementation requires relatively high thresholds in order to binarize the data and reduce its complexity prior to application of the edge-bundling method.

#### 2.3.4. Connexel data

Ultimately, our methods operate on connexel data. As described above, such data can be generated from full connectivity matrices in brainGL through thresholding. Independently from surfaces, it is possible to directly load connexel graphs for edge-bundling. Binary graphs can be loaded in the shape of a.fib file, a binary representation in VTK format normally used for the representation of fiber tracking results. To be interpreted as connexels, the.fib files may contain only lines with two points. Results of the edge-bundling process can be saved in the.fib format, which is supported by other software for the visualization of structural data such as DSI Studio^9^ and TrackVis^10^. brainGL also supports loading of weighted connexel graphs using a plain text file containing a tupel (*px py pz qx qy qz c*) of seven values on each line, separated by whitespace. *px, py* and *pz* are the 3D coordinates of one termination point *P* of the described connexel, *qx, qy* and *qz* the coordinates of the other termination point *Q*, and *c* is the connectivity between the two points. An example of a file containing three weighted connexels looks as follows:

125.3 12.0 31.1 145.2 34.3 25.6 0.61
146.1 25.9 54.2 135.3 24.4 25.2 0.12
156.2 32.8 22.7 154.3 34.5 45.5 0.76


#### 2.3.5. Scene configuration files and screenshots

The configuration of loaded datasets and their properties can be saved and loaded using scene files (.scn). Screenshots can be created using offscreen bitmap images with higher resolution than the screen (approximately 16000^2^ pixels, depending on the graphics card of the computer). The software also contains a flexible scripting system that allows for the animation of parameters and views through a series of screenshots.

### 2.4. Real-time display of seed-based connectivity

brainGL allows for interactive seed-point exploration of the connectivity on a cortical surface. After loading connectivity data, right-clicking on the surface displays connectivity values from that point. The colormap is user-defined and can be customized. The connectivity values are updated in real-time as the user selects or drags the cursor between nodes. This is similar to other real-time seed-point tools for the exploration of connectivity data (Cox, 1996; van Dixhoorn et al., 2010; Böttger et al., 2011; Saad and Reynolds, 2012).

### 2.5. Edge-bundling

**Figure 1:**
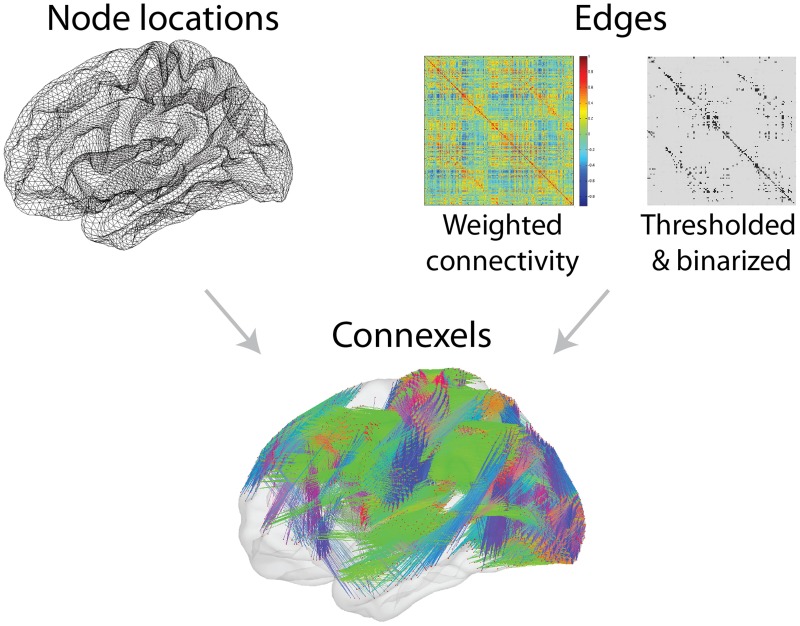
**Connectivity data can be described as connexels, six-dimensional pairs of three-dimensional spatial positions, and an associated connection strength**. As in this example of functional connectivity, such data can be represented with the node positions of a cortical surface (**Top left**), and a matrix of connection strengths (**Top right**). Connexels can be visualized with straight lines connecting each pair of connected nodes, but the structure of the data is unclear when a large number of connexels are included (**Bottom**).

As depicted in Figure 1, visualization of high-resolution connexel data with straight lines leads to a cluttered image in which the structure of the underlying data is not apparent. Edge-bundling algorithms were initially developed to improve the display of complex hierarchical graphs by grouping edges into bundles (Holten, 2006). Algorithms that can operate on arbitrary graphs have been introduced (Cui et al., 2008; Holten and van Wijk, 2009; Lambert et al., 2010; Telea and Ersoy, 2010; Ersoy et al., 2011; Gansner et al., 2011; Hurter et al., 2012) and used for three-dimensional data (Lambert et al., 2010). Our method for the bundling of connexels is inspired by Holten and van Wijk (2009) and Hurter et al. (2012). While Holten and van Wijk (2009) offer an algorithm that is extendable to three dimensions in a straightforward manner, it depends on several arbitrary parameters, and a numerical equilibrium of forces. This makes the results differ dramatically with changing data. Hurter et al. (2012) describe an algorithm that is numerically stable and independent from the density of the data. However, the sampling of the density contained in their method makes the extension to three-dimensional space infeasible. Our algorithm is described in more detail in Bottger et al. (2013), which shows that bundles with very different density bundle evenly with our mean-shift edge bundling.

The input is a set of binarized connexels, which can be derived from interactive exploration of weighted connectivity data, or loaded from a file as previously described in section 2.3.4. Next, a measure of similarity, termed *compatibility*, is calculated. The compatibility is a product of four geometrical criteria ranging from 1 for connexels with identical termination points to 0 for maximal dissimilarity. Following Holten and van Wijk (2009), for a connexel with termination points *P* and *Q*, these criteria are:

- Angle compatibility
  $$C_{a}(P,Q)=|\cos(\alpha )|$$
  with α: angle between the connexels
- Scale compatibility
  $$C_{s}(P,Q)=\frac{2}{l_{avg}\cdot \min\left(\left|P|,|Q\right|\right)+\max\left(\left|P|,|Q\right|\right)/l_{avg}}$$
  with $l_{avg}:\frac{\left|P|+|Q\right|}{2}$
- Position compatibility
  $$C_{p}(P,Q)=\frac{l_{avg}}{l_{avg}+||P_{m}-Q_{m}||}$$
  with *P*_*m*_, *Q*_*m*_: midpoints of connexels *P* and *Q*
- Visibility compatibility
  $$C_{v}(P,Q)=\min\text{​}\left(V(P,Q),V(Q,P)\right)$$
  with $V(P,Q):\max\text{​}\left(1-\frac{2||P_{m}-I_{m}||}{\left||I_{0}-I_{1}|\right|},0\right)$ and *I*_*m*_: midpoint of intersection points *I*_0_ and *I*_1_

**Figure 2:**
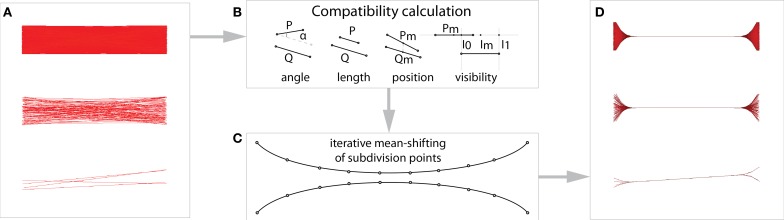
**(A)** Edge-bundling groups together geometrically similar connexels. **(B)** First, a measure of similarity (compatibility) between connexels is calculated from four geometrical criteria: length, angle, distance, and overlap (based on Holten and van Wijk, 2009). **(C)** Mean-shift edge-bundling then iteratively subdivides the connexels, and shifts compatible subdivision points toward their common mean. **(D)** Using a mean-shift has the advantage of bundling connexels with different density equally, and makes our method applicable to different datasets with the same default parameters.

A diagram of the different measures is shown in Figure 2. The overall compatibility *C*_*e*_ is defined as:

$$C_{e}\left(P,Q\right)=C_{a}\left(P,Q\right)\cdot C_{s}\left(P,Q\right)\cdot C_{p}\left(P,Q\right)\cdot C_{v}\left(P,Q\right)$$

The edges representing the connexels are then iteratively subdivided. The subdivision points with a compatibility value above a compatibility threshold *c*_thr_ are moved toward each other. The termination points remain fixed in their position. We use a scheme of 10 cycles consisting of 10 − *c* iterations, with *c* the number of the current cycle. We add equally spaced subdivision points along the edges between cycles by setting the number of segments to 1.3^*c*^.

To calculate the shifts of the subdivision points, we use the mean-shift algorithm (Fukunaga and Hostetler, 1975). This method estimates the density of points in the neighborhood of the subdivision points using a Gaussian kernel with a fixed radius *r*. Points are then iteratively moved toward areas of higher density by shifting them to the weighted average of all points in that neighborhood.

The bundling process visually groups compatible connexels into bundles, which share screen space in their midsection. This reduces the clutter inherent in the original visualization (Figure 2). We have shown that the combination of the concept of compatibility in connection with density estimation yields a stable bundling algorithm for connexel visualization (Bottger et al., 2013). The bundling results are largely independent from the data, and the use of the same parameters (*c*_thr_ = 0.8, *r* = 5 mm) yields convincing results for diverse datasets.

While edge-bundles improve the visual structure of the connections in a complex graph, the emphasis on the geometry of these connections makes it difficult to understand the spatial position of the termination points on the cortical surface. Edge-bundling also introduces ambiguity in the precise connection relations of the nodes because the curves run on top of each other in their midsections. The result is a visualization that clarifies the structure of dense bundles, but lacks a clear display of the connectivity patterns at the node-level.

### 2.6. Connectivity glyphs

The use of glyphs has previously been applied to diffusion weighted imaging (DWI) data (e.g., Basser et al., 1994; Tuch, 2004; Schultz and Kindlmann, 2010). For this application, glyphs encode the local diffusivity. However, the visualization of DWI data is fundamentally different from our pathless connectivity glyphs. Diffusion glyphs have the purpose of conveying the probability of anatomical paths at a given point in white matter, while our pathless connectivity glyphs are used to visualize connectivity patterns on the cortical surface.

We apply the use of glyphs to functional connectivity data by visualizing connectivity profiles in the form of glyphs at each connexel termination point on the cortical surface (*node*). Each glyph is a small visual summary of all connections from that node to the rest of the brain with a wide range of visualization parameters that can be manipulated by the user. This allows for the visualization of differences in connexels, either across large cortical areas, or at a local level between neighboring glyphs.

For the input of reduced data, the connectivity matrix is thresholded and binarized, typically resulting in several million connexels. Also, short connections below an interactively determined minimum length can be removed. To create each glyph, one point, line, or triangle for every remaining connexel is drawn. The orientation or strength of the connexel is used to derive the color of these display primitives.

For correct occlusion with the folded cortical surface, the glyphs are offset by one glyph radius in the camera viewing direction since otherwise the glyphs would appear mostly behind the cortical surface. The pie chart glyphs (described below) are further offset depending on their size, so that smaller pies are displayed on top of larger pies for multiple datasets.

#### 2.6.1. Glyph types

The user can choose between five different glyph types, which differ in the way the colored display primitives are spatially distributed. By representing full connectivity profiles in the very limited space of each node, each glyph type emphasizes various aspects of compactness, adherence to anatomy, or differences in connectivity between neighbors.

**Figure 3:**
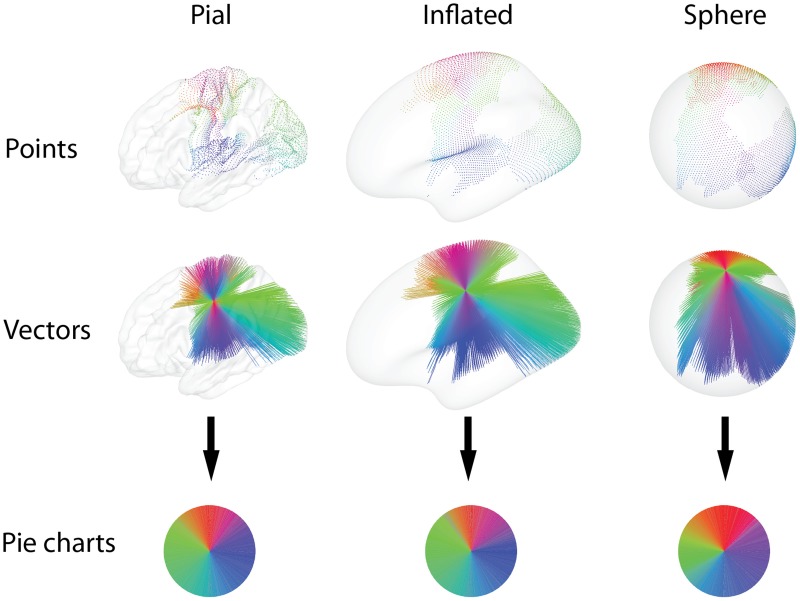
**Functional connectivity is calculated between each pair of nodes on a set of cortical surface representations, and thresholding yields a set of connections, here colored by orientation**. The colors and geometry of the connections are then used to calculate diverse glyph representations of the connectivity profile at each node. The vector and point glyph geometry is influenced by the choice of surface on a spectrum from the anatomically determined pial geometry, to the spherical representation. Drawing points diminishes overdraw, while drawing vectors emphasizes long-range connections. After sorting the colors by their hue, the pie charts emphasize the ratio of connections with different orientations.

**Figure 4:**
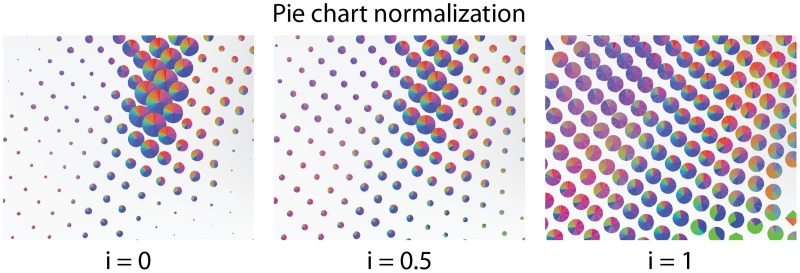
**The size of the pie charts is linearly interpolated between conveying the number of connected nodes **(Left)** and a constant radius **(Right)****.

**Figure 5:**
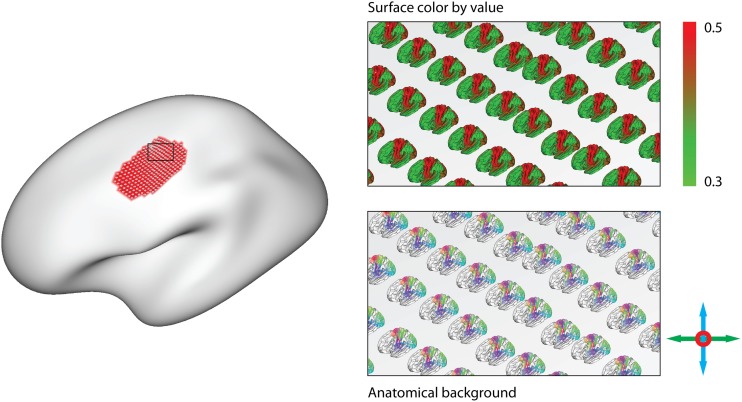
**After painting an area of interest (Left), anatomical background glyphs for an area-of-interest can be displayed**. They can either show color-mapped values on their surface (**Top right**), or serve to support the simplified glyphs (**Bottom right**).

**Figure 6:**
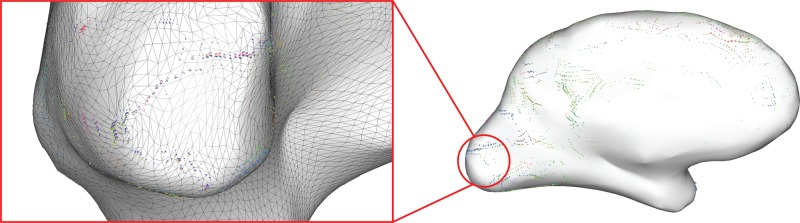
**Difference glyphs show the boundaries of the primary visual cortex (Left)**. The triangle mesh is overlaid to illustrate the placement of the difference glyphs in the middle of each triangle edge in order to show the difference in connectivity profile between two adjacent mesh nodes.

- Point glyphs: For each connection, we render a point *g*:
  $$g=p_{g}+s\cdot \left(p_{d}-q_{d}\right)$$
  *p*_*g*_ is the position of one termination point in the space that is used to render the underlying surface. *g* is shifted toward the other termination point by adding a three-dimensional offset. The offset is determined by scaling the relative position of the connected node using a *scaling factor s*.
  The result is a glyph on each node, consisting of points representing each connection from that node, and placing an emphasis on spatial information. We keep the spatial representations used for geometric offset (*p*_*d*_ and *q*_*d*_), color calculation, and glyph position (*p*_*g*_) independent. This makes it possible, for example, to draw glyphs shaped like the pial surface on the inflated surface while using the spherical representation to calculate the color. Additionally, it is possible to rotate the glyphs by three arbitrary angles around the principal axes. This allows for increasing the visibility of connected areas on the back of the glyph, which may otherwise be obscured.
- Vector glyphs: To better indicate the direction of connections, a line is drawn between each of the above mentioned points and the node position. This type of glyph representation emphasizes long-range connections (Figure 3) and makes it easy to identify changes in such connectivity between neighboring glyphs. The relative size of the point and vector glyphs is determined by the scaling factor *s*, which can be interactively adjusted to limit overlap with neighboring glyphs.
- Pie chart glyphs: To emphasize the distribution of orientations, each connection is represented through rendering of a triangular section in a small pie chart. The connections from each node are sorted according to the hue of their associated orientation colors (grouping connections in a similar direction) or connectivity value. This places the overall emphasis on color, and makes it possible to identify large cortical areas with strong connectivity in a certain direction, or with a certain distribution of connectivity values. During interactive manipulation of the viewpoint, the pie charts' orientations change so that they always face the viewer.
  For the pie charts, the radius *r* is determined by interpolating between the two following extreme cases: Setting the radius to *r*_*a*_ ∝ $\sqrt{n}$, with *n* being the number of connections, each connection is represented by an equally large area on the screen. The number of above-threshold connections from nodes can differ. This can lead to weakly connected nodes being very small, or strongly connected nodes overlapping with their neighbors. Using a constant radius *r*_*n*_ emphasizes the differences in ratio of different connections, but loses the valuable information about how connected a node is. We therefore allow for interactive interpolation between a constant radius and a radius proportional to the number of connected nodes by manipulating a parameter *i*. Afterwards, the radius *r* is calculated as *r* = *i* · *r*_*n*_ + (1 − *i*) · *r*_*a*_ (Figure 4).
- Anatomical background glyphs: The simplified glyphs are very efficient to render, and allow for a quick overview of whole-brain connectivity at interactive frame rates. However, it is difficult to see which precise anatomical areas contribute to the visible changes in connectivity between functional areas. To enable the investigation of such changes, *anatomical background glyphs* provide a complete depiction of the anatomical surface that scales and moves with the glyphs. Since rendering such a background for all nodes can easily overburden the graphics hardware, it is possible to limit the display of anatomical backgrounds to a specified area-of-interest. It is also possible to display color-coded connectivity maps on the anatomical background glyphs (Figure 5).
- Difference glyphs: To aid in the identification of transitions between adjacent cortical areas, the neighbor information inherent in the geometrical surface data is used to render *difference glyphs*. One glyph is created for each side of every triangle in the surface mesh, i.e., one glyph at the midpoint of each set of neighboring nodes. These glyphs display the differences between the connectivity profiles of the two nodes at the endpoints of the respective edge (Figure 6). The unthresholded connectivity values for each connexel are Fisher's r-to-z transformed and subtracted, and the absolute values of the differences are displayed after thresholding with the same methods previously described.

#### 2.6.2. Color

**Figure 7:**
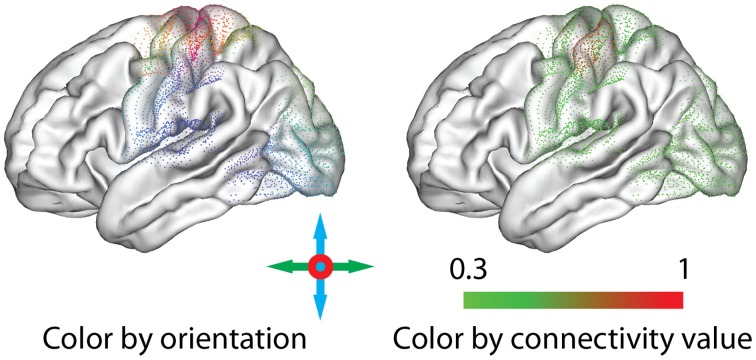
**Thresholded connectivity for an example point glyph, represented with different coloring options: the orientation of the connexel (Left) or the associated connectivity value (Right)**.

To emphasize different aspects of the available connectivity information, the application of two color schemes to the glyphs is possible (Figure 7):

- Orientation: To distinguish connections to different parts of the brain, colors can be assigned according to the orientation of each connection, similar to the standard scheme used for the visualization of DTI data (Douek et al., 1991). For each connected node, the connection vector is normalized, and the absolute value of the *x, y* and *z* components is used as red, green and blue, respectively. Colors can be assigned according to the orientation in a surface representation (i.e., pial, inflated, or sphere) independently from the placement of the glyphs or the calculation of the offsets.
- Value: To support comparison with connectivity value maps and provide additional information regarding the strength of connections, we assign colors to the display primitives using the same arbitrary color maps as for the seed-based real-time connectivity exploration (section 2.4). For the figures in this paper, we use an isoluminant green-to-red opponent-color scale, which is considered most appropriate for the display of values on a shaded surface (Borland and Taylor, 2007).

**Figure 8:**
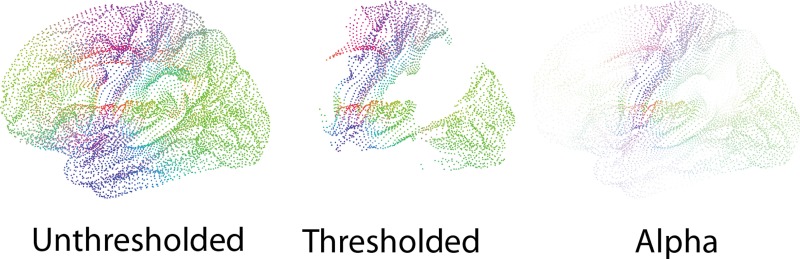
**Unthresholded (Left), thresholded (Middle) and transparent (Right) point glyphs**. Thresholding the glyphs leads to characteristic shapes, which also work well when minimized. Drawing glyphs with alpha blending makes it possible to perceive different connectivity values.

As an alternative to interactive thresholding, we enable manipulation of the transparency, or alpha value, of the display primitives. Display points can thus become gradually more transparent with smaller connectivity values (Figure 8).

## 3. Results

This section describes the results of the implementation of connectivity glyphs and edge-bundling in brainGL: After an introduction of the user interface, we present example applications for the visualization of functional connectivity and experiences regarding the runtimes and interactiveness of the resulting visualizations.

### 3.1. User interface

**Figure 9:**
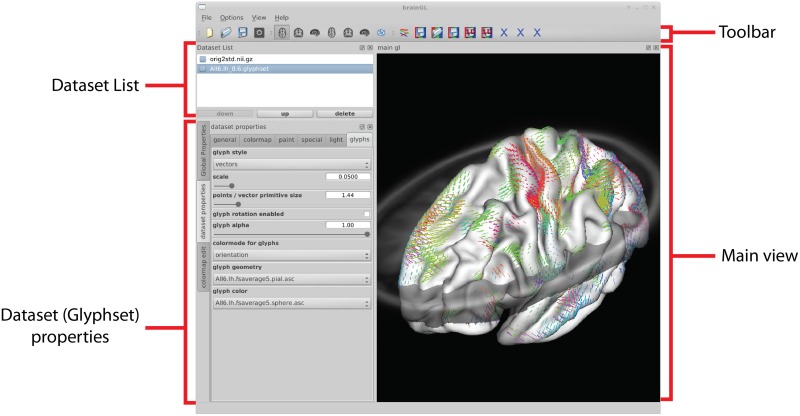
**The user interface is divided into a list of loaded datasets, three-dimensional views of the data, as well as global and dataset-specific properties**. Depending on which data type is currently selected, different buttons in the toolbar are made available.

The graphical user interface of brainGL is divided into several views, which can be freely arranged using the intuitive layout mechanisms of the GUI library. A typical configuration is shown in Figure 9. The loaded datasets are displayed in the *dataset list*, which also allows control over the visibility of the different datasets.

**Figure 10:**
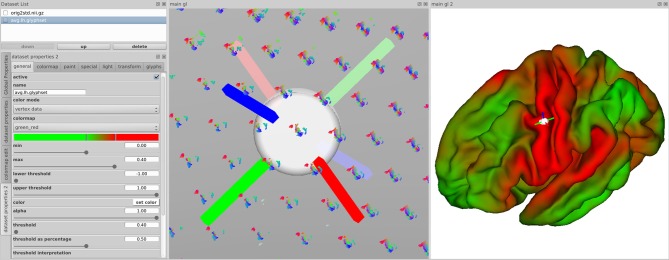
**Synchronized views of the data in brainGL showing visualization properties (Left), glyphs (Middle) and the color-mapped connectivity of a selected point (Right)**. Clicking on a node leads to the display of coordinate markers in both 3D views, and the display of the associated connectivity map in the right view. This allows for simultaneous overview of differences between neighboring nodes and their detailed individual connectivity.

The *main views* in brainGL are 3D renderings that support the interactive exploration of the data with standard techniques such as zooming, panning, and rotation. In addition to orthographic views, the software provides a simulated central perspective. Two coupled views with a synchronized selection cursor allow for multiple flexible application workflows. For example, while zoomed in to explore a single glyph visualization in detail, the second view can be used to gain an overview of where the selected glyph lies in the anatomical context (Figure 10).

Global parameters can either be shared between views or adjusted to different values. Owing to memory and performance limitations, glyph parameters are restricted to only one set of parameters for both views. Interaction with datasets in brainGL uses two mechanisms:

- Property panels: Properties of the selected dataset in the dataset list are accessible through property panels, which allow for manipulation of elementary data-types influencing the renderings. Each of these data-types is represented by a widget, for example, a scrollbar for float values. The glyph property panel (Figure 9, *left*) allows the user to influence the rendering of the connectivity glyphs. Most of the properties result in real-time feedback during a change. Only the selection boxes for the manipulation of the geometry and color require a recalculation of the large arrays of underlying display primitives. Changing these properties results in a delay before the resulting visualization can be explored. Global properties, such as the positioning of triplanar volume slices and colormaps used for the display of scalar information, can be customized in separate property panels.
- Toolbars: Operations on the datasets can be initiated from dataset-specific buttons that appear next to the standard toolbar depending on the type of data that is currently selected. The connectivity dataset toolbar features a button that allows for the creation of a dataset of connexels using the currently selected parameters (threshold, minimum length, geometry). Alternatively, connexels can be loaded from a file and then edge-bundled with a button in the connexel-dataset toolbar (using the algorithm described in section 2.5). Depending on the number of connexels in the dataset, this process may require long computation times, during which brainGL displays a progress bar. The bundles can then be explored in brainGL, or exported to other software for rendering.

### 3.2. Application examples

#### 3.2.1. Example datasets

The example dataset for connexel visualization is derived from functional connectivity as calculated from the following resting-state fMRI data. 65 participants (39 females, 26 males) between the ages of 11 and 83 years (mean age = 40.6 years, standard deviation = 19.6 years) from the enhanced Nathan Kline Institute–Rockland Sample^11^ were included. The datasets for each subject consisted of an anatomical scan, and fMRI measurements during rest.

The fMRI scans were recorded with the following parameters: *TR* = 645 ms, voxel dimensions 3 mm isotropic, 900 volumes. The preprocessing steps included: (1) discarding the first four EPI volumes from each resting-state scan, (2) motion correction, (3) slicetime correction, (4) time series despiking, (5) 4D mean-based intensity normalization, (6) removing linear trends, (7) regressing out eleven nuisance signals [six motion parameters and five top components from a principal components analysis of high variance signals (Behzadi et al., 2007; Chai et al., 2012)], and (8) band-pass temporal filtering (0.01-0.1 Hz). To reduce partial volume effects, no smoothing was performed.

The anatomical scans were co-registered with the functional data, and three spatial representations of the cortical surface were extracted using FreeSurfer (Dale et al., 1999; Fischl et al., 1999). The resulting pial, inflated, and spherical surface representations each consist of 10,242 corresponding nodes for each hemisphere in fsaverage5 space (Figure 3). FreeSurfer segments the cortex into two separate surfaces for the left and the right hemisphere. Since these surfaces spatially overlap in their inflated and spherical representations, a constant offset was applied between the two hemispheres, placing the surfaces close to each other without overlap.

Functional connectivity between pairs of nodes was calculated using Pearson correlation between the functional timeseries projected onto the surface, yielding 65 20484 × 20484 connectivity matrices. For the group data, the values were Fisher's r-to-z transformed, averaged, and transformed back with the inverse transform. This yielded an average connectivity matrix, which was included into a first glyphset for visualization in brainGL. In addition to the group average, correlations were calculated between age and connexel strength to show which connections change over the lifespan of the brain. The resulting matrix of r-values was included in a second glyphset.

#### 3.2.2. Example visualizations

We present here two examples to showcase the possibilities of connexel visualization using brainGL.

**Figure 11:**
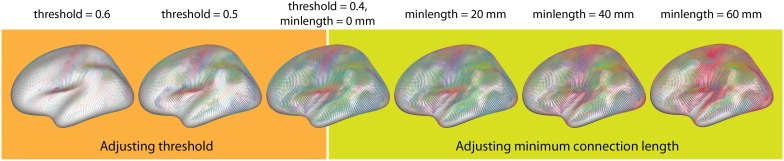
**Interactive exploration of the two free parameters of glyph visualization**. This example demonstrates the effects of adjusting the thresholding (**Left**) and removing short connections (**Right**).

**Figure 12:**
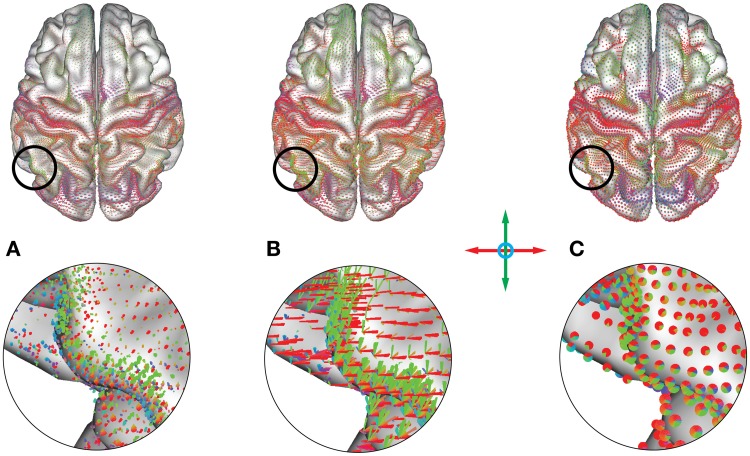
**(A)** Point, **(B)** vector and **(C)** pie chart glyphs for a whole-brain average functional connectivity dataset (threshold: *r* > 0.5, minimum length = 20 mm). Colors represent orientation of the underlying connections. The motor network presents as a red belt due to its lateral connectivity, and the visual network presents as collection of red/blue glyphs in the back of the brain. A multitude of other areas are distinguishable, representing a subdivision of the cortex into areas with similar functional connectivity profiles.

**Figure 13:**
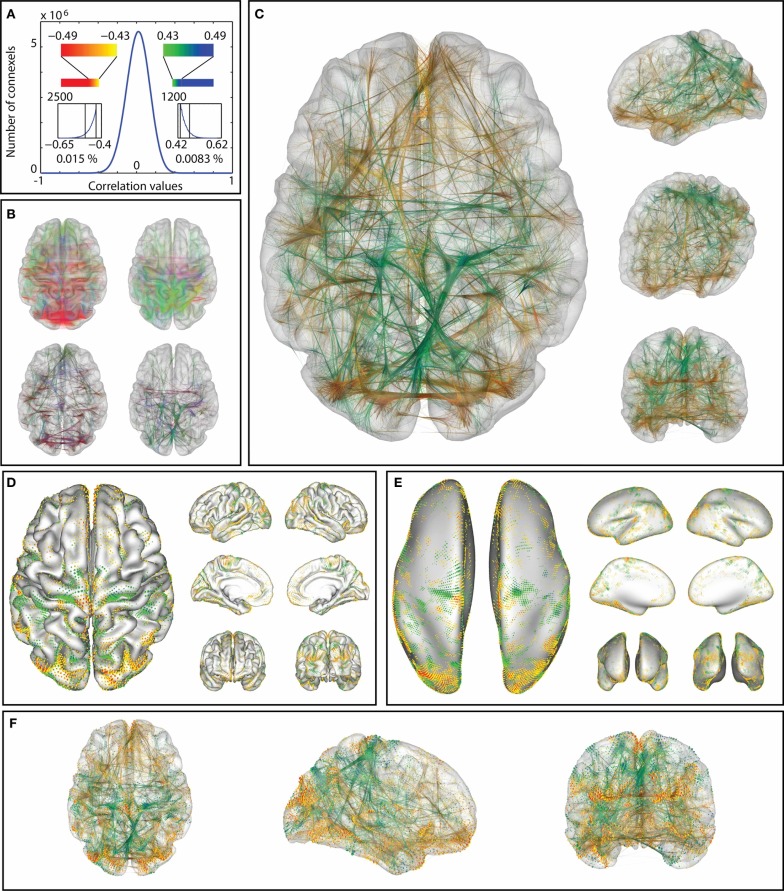
**Correlations between age and functional connectivity-based connexels in a group of 65 participants: the distribution of correlation values **(A)** is thresholded (at ±0.43, minimum length 20 mm)**. The remaining connections **(B)** vary strongly with age: The thresholded connexels in (b, right) gain connectivity strength over age, while the connexels in **(B** left) decrease in value (color represents orientation with xyz mapped to rgb). Edge-bundling **(B**, bottom, **C**) clarifies the structure of the connectivity graph. The same correlations visualized with surface connectivity glyphs **(D,E)**, which clarify the anatomical placement of the connections termination points on the pial **(D)** and inflated **(E)** surface representation. Combined visualization with glyphs and bundlings **(F)**. In **(C–F)**, positive and negative values are shown in the same visualization (yellow-to-red color scale for negative, green-to-blue color scale for positive correlation with age).

- Average connectivity: For the exploration of connectivity of the average dataset using glyphs, it was possible to load the dataset with a minimum threshold of 0.2, leaving 6.6·10^8^ connections (32%). The connectivity threshold and the minimum length for the removal of short edges were then interactively determined by optimizing the appearance of known connectivity networks. While varying the minimum length between 0 and 20 mm, additional details gradually appeared as shorter connections were omitted, thereby clarifying the connexel structure. For values higher than 20 mm, known functional connections started disappearing until only the longest connections remained.
  The threshold plays a similar role for the emphasis on stronger and removal of weaker connections. For values higher than 0.5, only the networks with the strongest connectivity (e.g., motor and visual connections) and strong local connections remain. Lowering the threshold results in the inclusion of more connections, which captures more subtle connectivity but also deemphasizes the stronger connections. Figure 11 illustrates the possibility of interactive variation of the two parameters during visual exploration.
  Figure 12 was created with a minimum length of 20 mm and a threshold of *r* > 0.5. Regardless of the chosen glyph type, the orientation color scheme makes it possible to immediately distinguish the largest networks in the brain, namely the motor and the visual cortex. The motor cortex appears as a laterally symmetrical belt of red connections in the central sulcus due to its strong interhemispheric connectivity with prominent left-right components. The visual cortex is clearly distinguishable in the occipital part of the brain by its strong red and blue colors. Other areas with strong connectivity in the anterior-posterior orientation (depicted in green) are also clearly distinguishable. Their structure becomes especially visible with higher minimum length thresholding using vector glyphs, which help to emphasize long-range connections.
- Correlation of connectivity with age: For the group dataset containing correlations between connexels and age, edge-bundling results and pie chart glyph visualizations are shown in Figure 13. The threshold was interactively adjusted to *r* > 0.43, and the minimum length to 20 mm to optimize the clarity of visible structure, while limiting the number of connexels to 40,000. After separate bundling of positively and negatively age-correlated connexels, the resulting bundlings were loaded simultaneously in brainGL, and the connexel values color-mapped to a green-blue (positive) and a yellow-red (negative) color scales. The screenshots of the bundlings (Figures 13B,C,F) were taken with a high resolution of 16,000 pixels in width.
  The utility of these visualizations is accentuated by the exploratory vantage they provide on the data. For example, the higher number of connexels decreasing versus increasing in strength with age has support from previous studies (Andrews-Hanna et al., 2007; Damoiseaux et al., 2008; Meunier et al., 2009), and could also be further investigated in the current dataset with statistical testing. The data also shows an apparent prevalence of age-related connexel decreases in frontal regions, and increases between posterior and central regions. Such observations could facilitate the generation of novel hypotheses, and provide the basis for subsequent statistical tests.

### 3.3. Complexity and efficiency

Our sample visualizations were created on an 8-core workstation with 3.4 GHz, 16 GB RAM, and a NVIDIA GeForce GTX 590 graphics adapter with 1.5 GB of RAM. The main memory is crucial for the bundling algorithm, since the compatibilities are precalculated between every pair of connexels. On the test system, the algorithm is therefore limited to 40,000 connexels. The bundling process for a set of connexels with that size took approximately 30 min.

For the glyphs, the limiting factor was the amount of graphics memory. Since the data was too complex to fit into the graphics memory as a whole, the data was restricted using minimum thresholds (see section 2.3) of 0.2 for the whole brain average connectivity and the age dependency dataset. For the pie charts, the additional sorting by color hue or value took several seconds after a change of parameters. With this exception, the use of graphics acceleration enabled interactive manipulation of viewpoint, thresholds, and scaling factor, even though the datasets contained several million connexels after thresholding.

## 4. Discussion

We have implemented *edge-bundling* and *connectivity glyphs* as two novel methods for the visualization of connexel data. These methods aim to display highly complex connectivity graphs without requiring reduction of the input data. Both methods are capable of displaying overlapping connexel structures embedded in the anatomical space, but each emphasizes unique aspects of the underlying data. Edge-bundling emphasizes the structure of the connections, showing high levels of common interconnections as bundles. Connectivity glyphs emphasize connectivity in relation to cortical anatomy. These method-specific qualities make edge-bundling more suited for illustration of connexel structure. Glyphs are more germane to anatomical localization of connexel patterns such as cortical mapping or presurgical planning.

### 4.1. Applications

While the application examples in this paper are derived from resting-state functional connectivity data, the methods themselves are modality-independent. They are equally applicable to any dataset describing connected nodes that are embedded in 3D space. In addition to the applications presented here of visualizing group-level functional connectivity and covariance with age, other applications may include the illustration of statistical group comparison data, pathological changes, or changes in connectivity structure over time. The increasing availability of modalities to measure pathless connection strengths between brain areas elicits an increasing need for tools to vizualize such information.

### 4.2. Implementation

The brainGL software provides a push-button implementation of the edge-bundling algorithm, which is achieved by the default settings of the two free parameters (compatibility threshold and radius of the mean-shift Gaussian kernel). The edge-bundling results are largely independent from the density of the connexel data (see Figure 2). brainGL also provides the possibility to deviate from the default parameters to enable individual adaption of the bundling results for data from drastically different applications or modalities.

In contrast to the plug-and-play implementation of edge-bundling—made possible largely by the robustness of the method against variation in the initial parameters—the utility of connectivity glyphs requires flexible interaction. Compromises between emphasizing certain aspects of the data are often necessary in order to convey information effectively. For example, vector glyphs are useful for identifying the presence or absence of particular features of an area's connectivity profile. Point glyphs are more useful for identifying subtle shifts in connectivity between adjacent regions. This is especially the case in combination with the anatomical background glyphs, which allow for the localization of such shifts in relation to the cortical morphology. The interactive optimization of the glyph parameters is made possible by the relatively low computational demand compared with the edge-bundling, which requires offline calculation.

As outlined in section 3.3, the glyphs rely mainly on the dedicated graphics hardware, while the bundling is performed on the central processing unit. The glyph visualizations consequently profit most from an advanced graphics card with multiple parallel stream processors and several gigabytes of graphics memory. For the bundling, the limiting factor in the current implementation is the amount of main memory.

The current brainGL implementation is restricted to datasets of ~10^5^ connexels for edge-bundling and ~10^8^ connexels for glyphs (using the hardware described in Section 3.3). For glyphs, high-performance graphics-dedicated systems may offer the processing capability to enable interactive manipulation of whole-brain, high-resolution connexel data. Edge-bundling, while also benefitting from high-performance hardware and improved memory use, will also profit from the implementation of multi-level bundling schemes (Gansner et al., 2011). Such bundling schemes start by iteratively grouping close connexels and then grouping groups of connexels on multiple levels, thus dramatically reducing the complexity.

### 4.3. Visualizing connexel uncertainty

The application of connexel methodology may have resisted general adoption due to a constellation of various analytic barriers to integrating the concept for brain research. The loss of signal in exploratory connexelwise analyses when applying classical conservative multiple comparison corrections such as Bonferroni (Nichols and Hayasaka, 2003) is one example. A limited set of less conservative methods have been developed (Worsley et al., 1998, 2005; Zalesky et al., 2012a), but remain far from widely adopted in the field. At this point, it is still an open question what assumptions can be made about connexel data, and what aspects of the data statistical selection methods can exploit. The computational complexity of dealing with connexels instead of voxels is another factor that makes the transfer of methods difficult. The application of edge-bundling and connectivity glyphs, however, is independent from the issue of thresholding. The visualization of connexels will profit from advances in statistical thresholding techniques independently from further development of rendering algorithms.

Alongside the need for improvement in connexel-specific statistical correction techniques, the visualization should also optimize the depiction of inherent uncertainty (Margulies et al., 2013). We have thus far targeted the need for high-resolution connectivity visualization at the expense of probability information. Especially in the case of glyphs, thresholding is integral to conveying differentiable patterns. Although the use of transparency and line contours has been effective for displaying subthreshold probability values in voxelwise visualizations (Allen et al., 2012), our initial integration of transparency into glyphs did not convey the necessary variance.

## 5. Conclusions

The visualization of high-resolution connexel datasets is of growing importance in brain research. Parallel to the development of analytic techniques, adaption and refinement of visualization practices are necessary. We offer edge-bundling and connectivity glyphs as two novel techniques. The continued development of similar interactive visualization software for connexel data will further provide a necessary foundation for mapping and understanding the connectome.

### Conflict of interest statement

The authors declare that the research was conducted in the absence of any commercial or financial relationships that could be construed as a potential conflict of interest.

## Footnotes

^1^code.google.com/p/braingl/

^2^qt.digia.com

^3^www.opengl.org

^4^glew.sourceforge.net

^5^www.vtk.org

^6^www.boost.org

^7^nifti.nimh.nih.gov

^8^surfer.nmr.mgh.harvard.edu

^9^dsi-studio.labsolver.org

^10^www.trackvis.org

^11^http://fcon_1000.projects.nitrc.org/indi/enhanced/

## References

[B1] Alexander-Bloch A. Giedd J. N. Bullmore E. (2013). Imaging structural co-variance between human brain regions. Nat. Rev. Neurosci. 14, 322–336 10.1038/nrn3465 23531697 PMC4043276

[B2] Allen E. Erhardt E. Calhoun V. (2012). Data visualization in the neurosciences: overcoming the curse of dimensionality. Neuron 74, 603–608 10.1016/j.neuron.2012.05.001 22632718 PMC4427844

[B3] Andrews-Hanna J. R. Snyder A. Z. Vincent J. L. Lustig C. Head D. Raichle M. E. (2007). Disruption of large-scale brain systems in advanced aging. Neuron 56, 924–935 10.1016/j.neuron.2007.10.038 18054866 PMC2709284

[B4] Basser P. J. Mattiello J. LeBihan D. (1994). MR diffusion tensor spectroscopy and imaging. Biophys. J. 66, 259–267 10.1016/S0006-3495(94)80775-1 8130344 PMC1275686

[B5] Beckmann C. F. DeLuca M. Devlin J. T. Smith S. M. (2005). Investigations into resting-state connectivity using independent component analysis. Philos. Trans. R. Soc. Lond. B Biol. Sci. 360, 1001–1013 10.1098/rstb.2005.1634 16087444 PMC1854918

[B6] Behzadi Y. Restom K. Liau J. Liu T. T. (2007). A component based noise correction method (CompCor) for BOLD and perfusion based fMRI. Neuroimage 37, 90–101 10.1016/j.neuroimage.2007.04.042 17560126 PMC2214855

[B7] Bernhardt B. C. Klimecki O. M. Leiberg S. Singer T. (2013). Structural covariance networks of the dorsal anterior insula predict females' individual differences in empathic responding. Cereb. Cortex [Epub ahead of print]. 10.1093/cercor/bht072 23535178

[B8] Biswal B. Yetkin F. Z. Haughton V. M. Hyde J. S. (1995). Functional connectivity in the motor cortex of resting human brain using echo-planar MRI. Magn. Reson. Med. 34, 537–541 10.1002/mrm.1910340409 8524021

[B9] Borland D. Taylor M. R. (2007). Rainbow color map (still) considered harmful. IEEE Comput. Graph. Appl. 27, 14–17 10.1109/MCG.2007.323435 17388198

[B10] Böttger J. Margulies D. S. Horn P. Thomale U. W. Podlipsky I. Shapira-Lichter I. (2011). A software tool for interactive exploration of intrinsic functional connectivity opens new perspectives for brain surgery. Acta Neurochir. 153, 1561–1572 10.1007/s00701-011-0985-6 21461877

[B11] Bottger J. Schafer A. Lohmann G. Villringer A. Margulies D. S. (2013). Three-dimensional mean-shift edge bundling for the visualization of functional connectivity in the brain. IEEE Trans. Vis. Comput. Graph. [Epub ahead of print]. 10.1109/TVCG.2013.114 23959625

[B12] Brookes M. J. Hale J. R. Zumer J. M. Stevenson C. M. Francis S. T. Barnes G. R. (2011). Measuring functional connectivity using MEG: methodology and comparison with fcMRI. Neuroimage 56, 1082–1104 10.1016/j.neuroimage.2011.02.054 21352925 PMC3224862

[B13] Bullmore E. Sporns O. (2009). Complex brain networks: graph theoretical analysis of structural and functional systems. Nat. Rev. Neurosci. 10, 186–198 10.1038/nrn2575 19190637

[B14] Chai X. J. Castanon A. N. Ongur D. Whitfield-Gabrieli S. (2012). Anticorrelations in resting state networks without global signal regression. Neuroimage 59, 1420–1428 10.1016/j.neuroimage.2011.08.048 21889994 PMC3230748

[B15] Cox R. W. (1996). Afni: software for analysis and visualization of functional magnetic resonance neuroimages. Comput. Biomed. Res. 29, 162–173 10.1006/cbmr.1996.0014 8812068

[B16] Cui W. Zhou H. Qu H. Wong P. C. Li X. (2008). Geometry-based edge clustering for graph visualization. IEEE Trans. Vis. Comput. Graph. 14, 1277–1284 10.1109/TVCG.2008.135 18988974

[B17] Dale A. M. Fischl B. Sereno M. I. (1999). Cortical surface-based analysis. I. segmentation and surface reconstruction. Neuroimage 9, 179–194 10.1006/nimg.1998.0395 9931268

[B18] Damoiseaux J. S. Beckmann C. F. Arigita E. J. Barkhof F. Scheltens P. Stam C. J. (2008). Reduced resting-state brain activity in the “default network” in normal aging. Cereb. Cortex 18, 1856–1864 10.1093/cercor/bhm207 18063564

[B19] Damoiseaux J. S. Rombouts S. A. Barkhof F. Scheltens P. Stam C. J. Smith S. M. (2006). Consistent resting-state networks across healthy subjects. Proc. Natl. Acad. Sci. U.S.A. 103, 13848–13853 10.1073/pnas.0601417103 16945915 PMC1564249

[B20] De Luca M. Beckmann C. F. De Stefano N. Matthews P. M. Smith S. M. (2006). fMRI resting state networks define distinct modes of long-distance interactions in the human brain. Neuroimage 29, 1359–1367 10.1016/j.neuroimage.2005.08.035 16260155

[B21] Douek P. Turner R. Pekar J. Patronas N. Le Bihan D. (1991). MR color mapping of myelin fiber orientation. J. Comput. Assist. Tomogr. 15, 923–929 10.1097/00004728-199111000-00003 1939769

[B22] Eklund A. Friman O. Andersson M. Knutsson H. (2011). A GPU accelerated interactive interface for exploratory functional connectivity analysis of fMRI data, in 2011 18th IEEE International Conference on Image Processing (ICIP), (Brussels), 1589–1592 10.1109/ICIP.2011.6115753

[B23] Ersoy O. Hurter C. Paulovich F. V. Cantareira G. Telea A. (2011). Skeleton-based edge bundling for graph visualization. IEEE Trans. Vis. Comput. Graph. 17, 2364–2373 10.1109/TVCG.2011.233 22034357

[B24] Fischl B. Sereno M. I. Dale A. M. (1999). Cortical surface-based analysis. II: inflation, flattening, and a surface-based coordinate system. Neuroimage 9, 195–207 10.1006/nimg.1998.0396 9931269

[B25] Fornito A. Zalesky A. Breakspear M. (2013). Graph analysis of the human connectome: promise, progress, and pitfalls. Neuroimage 80C, 426–444 10.1016/j.neuroimage.2013.04.087 23643999

[B26] Fukunaga K. Hostetler L. D. (1975). Estimation of gradient of a density-function, with applications in pattern-recognition. IEEE Trans. Inf. Theory 21, 32–40 10.1109/TIT.1975.1055330

[B27] Gansner E. R. Yifan H. North S. Scheidegger C. (2011). Multilevel agglomerative edge bundling for visualizing large graphs, in Pacific Visualization Symposium (PacificVis), 2011 IEEE, (Hong Kong), 187–194 10.1109/PACIFICVIS.2011.5742389

[B28] Holten D. (2006). Hierarchical edge bundles: visualization of adjacency relations in hierarchical data. IEEE Trans. Vis. Comput. Graph 12, 741–748 10.1109/TVCG.2006.147 17080795

[B29] Holten D. van Wijk J. J. (2009). Force-directed edge bundling for graph visualization. Comput. Graph. Forum 28, 983–990 10.1111/j.1467-8659.2009.01450.x22034356

[B30] Hurter C. Ersoy O. Telea A. (2012). Graph bundling by kernel density estimation. Comput. Graph. Forum 31, 865–874 10.1111/j.1467-8659.2012.03079.x

[B31] Irimia A. Chambers M. C. Torgerson C. M. Horn J. D. V. (2012). Circular representation of human cortical networks for subject and population-level connectomic visualization. Neuroimage 60, 1340–1351 10.1016/j.neuroimage.2012.01.107 22305988 PMC3594415

[B32] Johansen-Berg H. Behrens T. E. Robson M. D. Drobnjak I. Rushworth M. F. Brady J. M. (2004). Changes in connectivity profiles define functionally distinct regions in human medial frontal cortex. Proc. Natl. Acad. Sci. U.S.A. 101, 13335–13340 10.1073/pnas.0403743101 15340158 PMC516567

[B33] Lambert A. Bourqui R. Auber D. (2010). 3D edge bundling for geographical data visualization, in Information Visualisation (IV), 2010 14th International Conference, (London), 329–335 10.1109/IV.2010.53

[B34] Lerch J. P. Worsley K. Shaw P. W. Greenstein D. K. Lenroot R. K. Giedd J. (2006). Mapping anatomical correlations across cerebral cortex (MACACC) using cortical thickness from MRI. Neuroimage 31, 993–1003 10.1016/j.neuroimage.2006.01.042 16624590

[B35] Margulies D. S. Böttger J. Watanabe A. Gorgolewski K. J. (2013). Visualizing the human connectome. Neuroimage 80, 445–461 10.1016/j.neuroimage.2013.04.111 23660027

[B36] McGonigle J. Malizia A. L. Mirmehdi M. (2011). Visualizing functional connectivity in fMRI using hierarchical edge bundles, in Abstract and Poster at the 17th Annual Meeting of the Organization for Human Brain Mapping, (Quebec City).

[B37] Meunier D. Achard S. Morcom A. Bullmore E. (2009). Age-related changes in modular organization of human brain functional networks. Neuroimage 44, 715–723 10.1016/j.neuroimage.2008.09.062 19027073

[B38] Nichols T. Hayasaka S. (2003). Controlling the familywise error rate in functional neuroimaging: a comparative review. Stat. Methods Med. Res. 12, 419–446 10.1191/0962280203sm341ra 14599004

[B39] Saad Z. S. Reynolds R. C. (2012). Suma. Neuroimage 62, 768–773 10.1016/j.neuroimage.2011.09.016 21945692 PMC3260385

[B40] Schultz T. Kindlmann G. L. (2010). Superquadric glyphs for symmetric second-order tensors. IEEE Trans. Vis. Comput. Graph. 16, 1595–1604 10.1109/TVCG.2010.199 20975202

[B41] Sporns O. (2013). Making sense of brain network data. Nat. Methods 10, 491–493 10.1038/nmeth.2485 23722207

[B42] Telea A. Ersoy O. (2010). Image-based edge bundles: simplified visualization of large graphs. Comput. Graph. Forum 29, 843–852 10.1111/j.1467-8659.2009.01680.x

[B43] Tuch D. S. (2004). Q-ball imaging. Magn. Reson. Med. 52, 1358–1372 10.1002/mrm.20279 15562495

[B44] van Dixhoorn A. F. Milles J. van Lew B. Botha C. P. (2012). BrainCove: A tool for voxel-wise fMRI brain connectivity visualization, in Eurographics Workshop on Visual Computing for Biology and Medicine, (Norrköping), 99–106

[B45] van Dixhoorn A. Vissers B. Ferrarini L. Milles J. Botha C. (2010). Visual analysis of integrated resting state functional brain connectivity and anatomy, in Proceedings of the 2nd Eurographics Conference on Visual Computing for Biology and Medicine (EG VCBM'10), (Leipzig), 57–64

[B46] Worsley K. J. Cao J. Paus T. Petrides M. Evans A. C. (1998). Applications of random field theory to functional connectivity. Hum. Brain Mapp. 6, 364–367 10.1002/(SICI)1097-0193(1998)6:5/6<364::AID-HBM6>3.0.CO;2-T 9788073 PMC6873373

[B47] Worsley K. J. Chen J.-I. Lerch J. Evans A. C. (2005). Comparing functional connectivity via thresholding correlations and singular value decomposition. Philos. Trans. R. Soc. Lond. B Biol. Sci. 360, 913–920 10.1098/rstb.2005.1637 16087436 PMC1854921

[B48] Xia M. Wang J. He Y. (2013). Brainnet viewer: a network visualization tool for human brain connectomics. PLoS ONE 8:e68910 10.1371/journal.pone.0068910 23861951 PMC3701683

[B49] Zalesky A. Cocchi L. Fornito A. Murray M. M. Bullmore E. (2012a). Connectivity differences in brain networks. Neuroimage 60, 1055–1062 10.1016/j.neuroimage.2012.01.068 22273567

[B50] Zalesky A. Fornito A. Egan G. F. Pantelis C. Bullmore E. T. (2012b). The relationship between regional and inter-regional functional connectivity deficits in schizophrenia. Hum. Brain Mapp. 33, 2535–2549 10.1002/hbm.21379 21922601 PMC6870162

[B51] Zalesky A. Fornito A. Bullmore E. T. (2010). Network-based statistic: identifying differences in brain networks. Neuroimage 53, 1197–1207 10.1016/j.neuroimage.2010.06.041 20600983

[B52] Zuo X. N. Ehmke R. Mennes M. Imperati D. Castellanos F. X. Sporns O. (2012). Network centrality in the human functional connectome. Cereb. Cortex 22, 1862–1875 10.1093/cercor/bhr269 21968567

